# Structure of the Anti-C60 Fullerene Antibody Fab Fragment: Structural Determinants of Fullerene Binding

**Published:** 2019

**Authors:** E. M. Osipov, O. D. Hendrickson, T. V. Tikhonova, A. V. Zherdev, O. N. Solopova, P. G. Sveshnikov, B. B. Dzantiev, V. O. Popov

**Affiliations:** Bach Institute of Biochemistry, Research Center of Biotechnology of the Russian Academy of Sciences, Leninsky Ave. 33, 119071, Moscow, Russia; Russian Research Center of Molecular Diagnostics and Therapy, Simpheropolsky Blvd. 8, 113149, Moscow, Russia

**Keywords:** antibodies, fullerene, molecular modeling, X-ray analysis

## Abstract

The structure of the anti-C_60_ fullerene antibody Fab fragment (FabC_60_) was solved by X-ray crystallography. The computer-aided docking of C_60_ into the antigen-binding pocket of FabC_60_ showed that binding of C_60_ to FabC_60_ is governed by the enthalpy and entropy; namely, by π-π stacking interactions with aromatic residues of the antigen-binding site and reduction of the solvent-accessible area of the hydrophobic surface of C_60_. A fragment of the mobile CDR H3 loop located on the surface of FabC_60_ interferes with C_60_ binding in the antigen-binding site, thereby resulting in low antibody affinity for C_60_. The structure of apo-FabC_60_ has been deposited with pdbid 6H3H.

## INTRODUCTION

The problem of immune recognition is one of the main challenges of modern biochemistry, important both for understanding biological processes and for designing new drugs and vaccines. A considerable body of theoretical and experimental data accumulated in recent years provides deeper understanding of the structural and functional patterns of immune interactions [1-5]. X-ray crystallography is among the most powerful methods used to study the three-dimensional structures of specific antibody–antigen complexes and gather detailed insights into the interactions of antibodies with various high-molecular-weight antigens (proteins, polysaccharides, lipids, etc.) and water-soluble low-molecular-weight haptens [6-9].

In recent years, there has been a significant expansion of the range of potential targets for immune recognition, in particular due to particles with a structurally degenerate surface. This class includes engineered nanoparticles (ENPs) that are characterized by a growing production and applications in various fields of science and technology [10]. The opportunity to manipulate the physicochemical parameters of nanoparticles opens new prospects for the synthesis of nanoparticles with desired properties for application in targeted drug delivery, disease diagnosis, imaging of organs and tissues, etc. [11-13]. The use of ENPs in medicine and biotechnology raises the question of their immunogenic properties.

Antigens that do not fit into the standard patterns of the immune reaction include fullerenes: nanoparticles consisting exclusively of carbon atoms and characterized by a unique geometry and properties [14]. A number of studies provide evidence of the possible formation of fullerene-specific antibodies [15-18].

The structure of the fullerene-binding site of antibodies was considered in the only study [15]. Using X-ray crystallography and computer simulation, the specific fullerene-binding site was shown to be a spherical cavity 7 Å in diameter that is formed by a cluster of hydrophobic amino acids. However, in the structural model of the fullerene-Fab complex [15], hydrophobic residues outside the CDR are included in the interaction with fullerene.

The aim of this investigation is to study the structural parameters of epitopes that specifically recognize insoluble antigens and elucidate the characteristic features of the formation of appropriate immune complexes by X-ray analysis and molecular modeling of the Fab-fullerene complex. We used the Fab fragment (FabC_60_) of the previously obtained monoclonal antibody to C_60_ fullerene [18].

## EXPERIMENTAL

**Materials**

The soluble form of C_60_, fullerene aminocaproic acid (C_60_(H)3(NH(CH_2_)_5_COONa)3 × 10 H_2_O) (SolC_60_, 98% purity), was purchased from Intelfarm (Russia). Peroxidase-labeled goat anti-mouse lambda light chain antibodies were purchased from Bethyl Laboratories, Inc. (USA). 3,3´,5,5´-Tetramethylbenzidine (TMB) and Triton X-100 were purchased from Sigma-Aldrich (USA). Other reactants and buffer components were of analytical grade.

For the ELISA, Costar 9018 microplates (Corning, USA) were used.

**Production of mouse monoclonal Fab fragments**

In this study, we used the Fab fragment of clone B1 of mouse monoclonal antibody (Ful B1, IgG2a lambda) obtained in our previous work [18]. The antibody Ful B1 was purified from ascitic fluid using one-step protein G–Sepharose affinity chromatography and dialyzed overnight against 200 mM sodium phosphate buffer, pH 7.4, containing 2 mM EDTA and 10 mM cysteine. To obtain Fab fragments of Ful B1 (FabC_60_), papain (2x crystallized from Papaya Latex, Sigma, USA) was dissolved in the same buffer, mixed with the antibody solution at a 1 : 100 ratio, and incubated for 4 h at 37 °C with gentle shaking. The digestion was stopped by adding iodoacetic acid to a final concentration of 10 mM. To remove the Fc fragments, the reaction mixture was applied onto a Protein A Sepharose column and the flow-through was collected and dialyzed against PBS. The FabC_60_ fragment concentration was determined by spectrophotometry at 280 nm using E (1 mg/ml) = 1.4. The purity of the samples was assessed using 12 % SDS-PAGE.

**Characterization of mouse monoclonal Fab fragments**

*Indirect ELISA. *The C_60_–TG immunoconjugate (5 μg/ mL) in PBS was added to microplate wells and incubated for 16 h at 4°C. The plate was washed four times with PBS supplemented with 0.05% Triton X-100 (PBST). Then, a series of dilutions of the Ful B1 antibody and its FabC_60_ fragment in PBST were added to the microplate wells and incubated for 1 h at 37°C. After washing the microplate, peroxidase-labeled goat anti-mouse lambda light chain antibodies were added to the wells (1:10,000 dilution of the commercial preparation) and incubated for 1 h at 37°C. After the final washing, the peroxidase activity of the resulting complexes was measured. For this purpose, a substrate solution containing 0.42 mM TMB and 1.8 mM hydrogen peroxide in 0.1 M sodium citrate buffer, pH 4.0, was added to each microplate well and the incubation was carried out for 15 min at room temperature. The enzymatic reaction was terminated by adding 50 μL of 1 M H_2_SO_4_ to each well. The optical density of the oxidation product was measured at 450 nm using a Zenyth 3100 microplate photometer (Anthos Labtec Instruments, Austria).

*Competitive ELISA of SolC_60_ using Fab fragments. *To detect SolC_60_, the C_60_–STI conjugate (1 μg/mL) in PBS was added to microplate wells and incubated for 16 h at 4°C. The plate was washed four times with PBST. After that, a series of dilutions of SolC_60_ (from 5 μg/mL to 0.1 ng/mL) and FabC_60_ at a concentration of 5 μg/mL were added to the microplate wells and the microplate was incubated for 90 min at 37°C. After washing, peroxidase-labeled goat anti-mouse lambda light chain antibodies were added to the wells (1:10,000 dilution of the commercial preparation) and the microplate was incubated for 1 h at 37°C. After the final washing, the peroxidase activity of the resulting complexes was measured as described above.

The plots of optical density (*y*) versus antigen concentration in the sample (*x*) were fitted to a four-parameter logistic function using the Origin 7.5 software (OriginLab, USA):

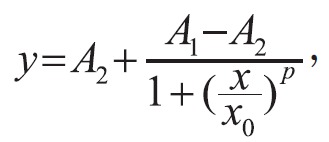



where *A1 *is the maximum signal, *A2 *is the minimum signal, *p *is the slope of the calibration curve, and *x0 *is the antigen concentration causing 50% inhibition of antibody binding (*IC_50_*).

**Crystallization**

Two protein solutions were used for crystallization: a solution of FabC_60_ (7 mg/ml) in 50 mM HEPES, pH 7.0, and a solution of the FabC_60_ complex with SolC_60_. The complex was prepared by mixing 100 μL of the 0.16 mM (7 mg/ml) FabC_60_ solution with 20 μL of a 1 mM SolC_60_ solution in water.

For both protein solutions, crystallization conditions were screened and optimized using the hanging-drop vapor-diffusion technique at 298 K. Screening was performed using crystallization screens Index HR2-134 and Crystal Screen HR2-110/112 (Hampton Research, USA). The drops were composed of equal volumes (1 μL) of the protein and reservoir solutions. FabC_60_ crystals suitable for the diffraction experiments were obtained using the following conditions: 25 % w/v PEG 3350, 0.2 M (NH_4_)_2_SO_4_, 0.1 M Bis-Tris, pH 6.5. The crystals appeared on the third day and grew to a maximum size of 200×200×50 μm within a week.

**X-ray data collection and structure determination**

The X-ray data set was collected from a FabC_60_ crystal on the K4.4e beamline at the Belok station for protein crystallography at the Kurchatov synchrotron-radiation source (Moscow, Russia) at a wavelength of 0.98 Å equipped with a Rayonix SX165 CCD detector at 100 K under nitrogen flow. Prior to data collection, the crystal was soaked in the reservoir solution supplemented with 20 % v/v glycerol and then flash-cooled in liquid nitrogen. The X-ray data were processed and merged with XDS [19]. The crystallographic calculations were performed using the CCP4 suite of programs [20]. The FabC_60_ structure was solved by the molecular replacement method with the BALBES pipeline [21]. The structure with the PDB ID 1MFB [22] was the best scoring search model. The structure was refined with REFMAC5 [23]. Visual inspection and manual rebuilding of the model were performed with COOT [24]. Data collection and structure solution statistics are summarized in *Table*. The figures were prepared using PyMOL [25]. The structure was deposited with the Protein Data Bank (PDB entry 6H3H).

**Table:** Statistics of data collection and structure refinement

Data collection	
Space group	P2_1_
Unit cell parameters a, b, c(Å), β (o)	40.18;137.58;83.15 91.9
Resolution	28.83-1.91 (2.02-1.91)
I / σ	17.5 (3.2)
Completeness (%)	99.5 (97.3)
Total reflections	349716 (52972)
Unique reflections	69665 (10963)
Multiplicity	5.0 (4.8)
*R_meas_ (%)	8.4 (2.3)
CC_1/2_	99.9 (83.0)
Wilson plot B-factor	29.7
Refinement	
R_cryst_ (%)	19.3 (27.9)
R_free_ (%)	23.5 (34.3)
Bond r.m.s.d. from ideal values:	
Length (Å)	0.02
Angle (o)	1.9
Torsion angle (o)	7.2
Number of atoms	
Protein	6535
Water	456
Average B-factors (Å^2^)	
Protein	30.1
Water	32.5
Statistics of Ramachandran plot	
Allowed region(%)	97.4
Disallowed region(%)	0.2

^*^For R_meas_, the value in parentheses is given for the inner shell.

**Small-molecule docking**

Docking and preparation of the receptor/ligand structures were performed in Autodock Vina [26] implemented in Pymol-Script-repo (https://github.com/Pymol-Scripts/Pymol-script-repo). The coordinates for C_60_ were derived from ChemSpider (http://www.chemspider.com). The receptor grid for docking in FabC_60_ was defined as a box with a side of 22.5 Å, the center at (13.95; -9.51; 38.74), and 60 grid points in each dimension.

## RESULTS AND DISCUSSION

**Characterization of mouse monoclonal Fab fragments**

**Fig. 1:**
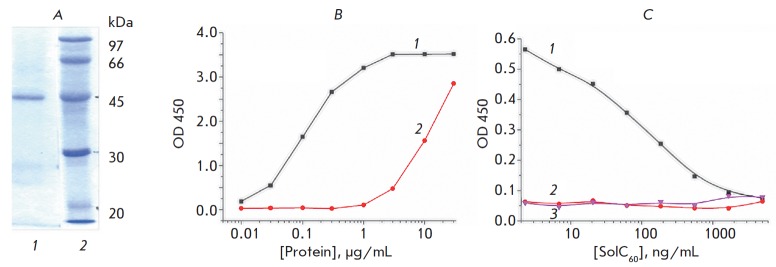
*A *– 12% SDS-PAGE of FabC_60_; lane *1*, FabC_60_; lane *2*, molecular weight markers (phosphorylase B, 97 kDa; bovine serum albumin, 66 kDa; ovalbumin, 45 kDa; carbonic anhydrase, 30 kDa; trypsin inhibitor, 20 kDa). *B *– Titration curves for Ful B1 monoclonal antibody (*1*) and FabC_60_ (*2*) in the indirect ELISA. *C *– Competitive ELISA curves for SolC_60_ obtained using immobilized C_60_–STI and FabC_60_ (*1*), immobilized C_60_–STI and Fab fragment of monoclonal antibody against potato virus X (*2*), and immobilized atrazine–STI and FabC_60_ (*3*)

FabC_60_ used in the present work were produced by papain digestion of the full-size anti-C_60_ fullerene mouse monoclonal antibody Ful B1 and purified to a homogeneous state, which was confirmed by 12% SDS-PAGE under non-reducing conditions (*Fig. 1A*).

The immune reactivity of the obtained FabC_60_ was assessed by indirect ELISA using immobilized C_60_–TG immunoconjugate (*Fig. 1B*). Indirect ELISA showed that the immunoreactivity of FabC_60_ was ~ 80 times lower than that of the full-size antibody.

**Fig. 2:**
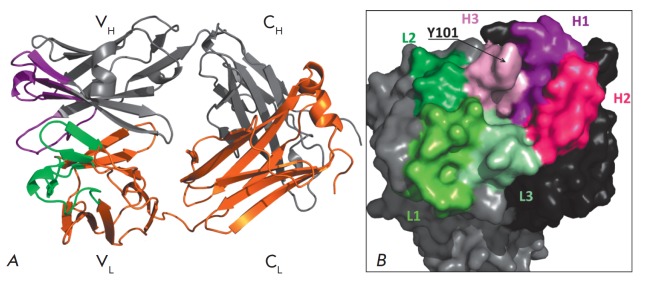
*A *– FabC_60_ structure. The H chain and its CDR are shown in gray and violet, respectively; the L chain and its CDR, in orange and green, respectively. *B *– The surface of the antigen-binding pocket in FabC_60_ viewed approximately along the largest dimension of the FabC_60_ molecule. The surfaces of the H and L chains are shown in black and gray, respectively. The surfaces of the H and L CDRs are depicted by shades of purple and green, respectively

The antigen-binding capacity of FabC_60_ was characterized by competitive ELISA. In this assay, the C_60_–protein conjugate adsorbed on the solid phase and the water-soluble fullerene derivative, SolC_60_, competitively interacted with FabC_60_. The Fab fragment of monoclonal antibody to another antigen (potato virus X), as well as a protein conjugate with another hapten (pesticide atrazine) immobilized on the solid phase, was used as control to confirm the specificity of interaction (*Fig. 1C*). As can be seen in *Fig. 1C*, FabC_60_ does not interact with the adsorbed atrazine–protein conjugate (curve 3). In addition, the immobilized C_60_–STI showed no binding to non-specific antibodies (curve 2 in *Fig. 1C*). Curve 1 in *Fig. 1C* demonstrates the competition between free SolC_60_ in solution and the adsorbed C_60_– STI conjugate for the binding sites of the antibody. All these effects confirm the specific nature of the immune interaction.

**FabC_60_ structure**

Crystallization of the FabC_60_ complex with SolC_60_ yielded crystals of the free form of FabC_60_. Since the structures of FabC_60_ obtained in the presence and absence of SolC_60_ were identical, hereinafter we will consider only the data set collected from the crystals of the free form of FabC_60_.

The FabC_60_ structure was solved by X-ray crystallography at a 1.9 Å resolution. Data collection and refinement are summarized in *Table*. There are two FabC_60_ molecules per asymmetric unit. The RMSD between 2,717 equivalent atoms upon superposition of these FabC_60_ molecules is 1.0 Å. When superimposed by Cα atoms of the variable domain, RMSD was 0.4 Å. Two crystallographically independent molecules differ in the conformation of the C-terminus and in intermolecular contacts (see below).

FabC_60_ has a two-domain structure typical of Fab fragments, consists of heavy (H) and light (L) λ chains, and has the dimensions 45×50×100 Å (*Fig. 2A*). The variable domain of the heavy chain (V_H_) includes residues 1–119, and the variable domain of the light chain (V_L_) includes residues 1–108. The constant CH domain includes residues 123–222, and the C_L_ domain includes residues 115–215. All the amino acid residues of the light chains are seen on electron density maps. The residues 135–139 located in the loop between the V_H_ and C_H_ domains are disordered and are not visible on electron density maps.

The complementarity-determining regions (CDRs) are defined as follows: **L1 **(Arg23–Asn36), **L2 **(Gly51–Ala57), **L3 **(Ala91-Val99), **H1 **(Gly26– His35), **H2 **(Tyr50–Glu59), and **H3 **(Gly99-Trp109) [27, 28] (*Fig. 2B*). The residues in these regions form an antigen-binding pocket with the sizes 9×7 Å and a depth of 5 Å (the diameter of C_60_ is 7 Å). Taking into account the presence of the π–π conjugated system in fullerene, we expected the CDR to contain aromatic residues prone to π–π stacking interactions. Indeed, the surface of the antigen-binding pocket is partially formed by aromatic residues located in CDR – Tyr50 (H2), Tyr101 (H3), Tyr34 (L1), Trp93 (L3), and Trp98 (L3). The side chain of Tyr101 of the Asp100–Tyr101 loop in CDR H3 is poorly seen on electron density maps, and the B-factors of the side-chain atoms of Tyr101 (B_mean_= 54 Å2) are higher than those of the main-chain atoms of Tyr101 (B_mean_= 28 Å2). This difference in the B-factors indicates the mobility of the side chain of Tyr101 and may be associated with the location of Tyr101 on the protein surface. The mobile Tyr101 can act as a lid of the pocket, thereby hindering the access of C_60_ to the antigen-binding pocket and reducing the affinity of the antibody-antigen interaction (*Fig. 2B*).

**Fig. 3:**
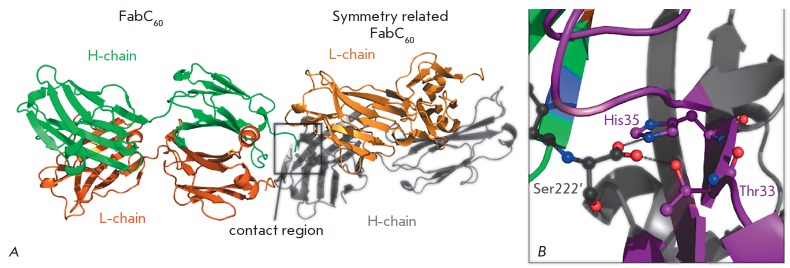
*A* – Crystal packing of FabC_60_ in space group P21. The C-terminus of the H chain (green) of FabC_60_ protrudes into the antigen-binding site of the symmetry-related FabC_60_. *B *– The contact region (the orientation and coloring differ from those in *Fig. 3a*). Binding of C-terminal Ser222’ of the H chain of the symmetry-related molecule in the antigen-binding pocket of FabC_60_. The CDRs of the H and L chains are shown in purple and green, respectively. The hydrogen bonds of the carboxyl group of C-terminal Ser222’ (carbon atoms being shown in black) are indicated by black dashed lines

The residues Thr33 and His35 of CDR H1 on the surface of the antigen-binding site can form hydrogen bonds that can be involved in fullerene binding [15]. However, in the FabC_60_ free-form structure, the residues Thr33 and His35 of the FabC_60_ molecules formed hydrogen bonds with the C-termini of the H or L chain of the symmetry-related FabC_60_ (*Fig. 3A,B*). In one crystallographically independent FabC_60_ molecule, the antigen-binding pocket was occupied by the C-terminal peptide Ala219–Ser222 of the H chain of the symmetry-related molecule, the carboxyl group of Ser222 forming hydrogen bonds with Thr33 and His35. The antigen-binding pocket of the second crystallographically independent FabC_60_ molecule was occupied by the C-terminal peptide Asp213–Ser215 of the L chain of another symmetry-related FabC_60_ molecule. In this case, the hydrogen bonds of C-terminal Ser215 with the residues Thr33 and His35 of the H chain occured via a water molecule. Most likely, the binding of the C-terminus of one FabC_60_ molecule in the antigen-binding pocket of another molecule was an artifact of the crystal packing and is unrelated to the biological role of FabC_60_. However, this crystal packing stabilized by additional intermolecular hydrogen bonds with CDR apparently hinders the antigen binding, which may be responsible for the unsuccessful attempts to crystallize FabC_60_ in complex with SolC_60_.

**Fig. 4:**
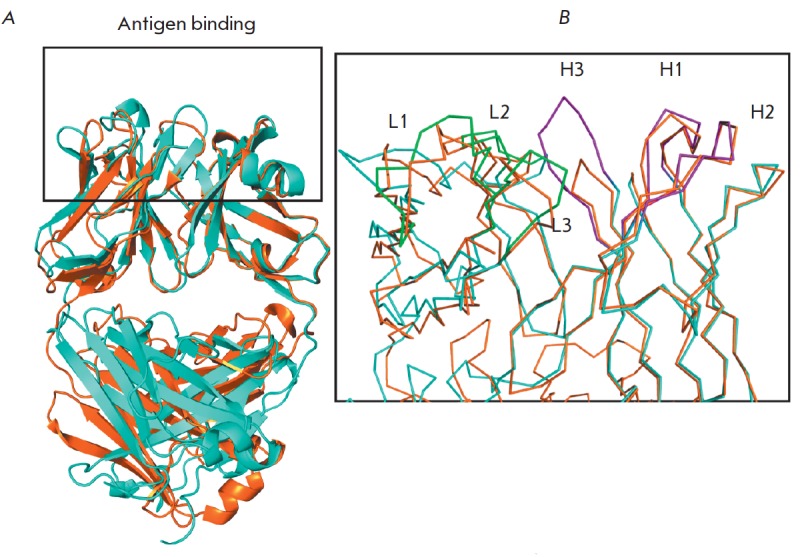
*A *– Structures of FabC_60_ (cyan) and anti-C_60_ Fab (orange) [15] superimposed on the VH domains. The antigen-binding pocket is highlighted by a black box. *B *– The zoomed antigen-binding pockets of FabC_60_ (cyan) and anti-C_60_ Fab (orange) superimposed on the VH domains. The CDRs of the H and L chains are shown in purple and green for FabC_60_. Note the difference in the length of CDR H3

The degree of homology between the primary structures of the FabC_60_ and Fab fragments of anti-fullerene antibodies that had been structurally characterized earlier [15] was rather high and amounted to 76% and 40% for the H and L chains, respectively. FabC_60_ contains the λ-chain, while Fab fragments of anti-fullerene antibodies contained the κ-chain. However, the structures of antibodies are dissimilar due to the different mutual arrangements of the V- and C-domains (RMSD > 3 Å). Therefore, it is difficult to perform a comparative analysis of these two structures. When superimposed by the VH domains, RMSD is 0.4 Å (*Fig. 4A*). The antigen-binding pockets of these two antibodies also differ in their composition and structure; the maximum difference is observed in CDR H3 and the L-chain fold (*Fig. 4B*). The H3 loop of the Fab fragment described by Braden et al. [15] is seven residues shorter (four residues) than that in FabC_60_ (11 residues).

**Docking of C_60_ into FabC_60_ and analysis of C_60_ binding to FabC_60_**

**Fig. 5:**
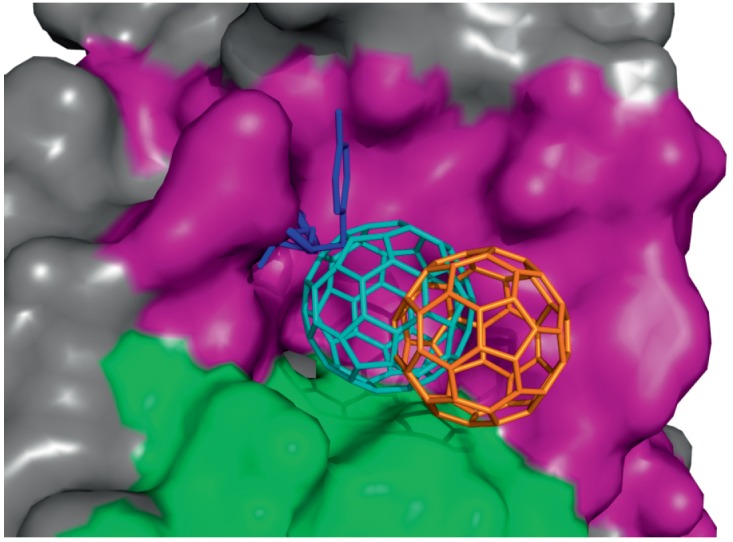
Docking of C_60_ (represented by the stick model) to the unmodified (orange C_60_) and modified (cyan C_60_) antigen-binding pockets of FabC_60_. The residues Asp100– Tyr101 that are absent in the modified structure are represented by the blue stick model. The surfaces of the H and L chains of the modified antigen-binding pocket used to generate the receptor in Autogrid are shown in purple and green, respectively

Since we failed to obtain a structure of FabC_60_ in complex with C_60_, we performed rigid body docking of C_60_ into the antigen-binding pocket of FabC_60_ [26] in order to elucidate the structural features of the antigen-binding site of anti-fullerene antibodies. The antigen binding is known to be accompanied by conformational changes within CDRs [29, 30]. The largest structural rearrangements are observed in the most mobile CDR H3 loop, which is the one most difficult to simulate as compared to all other loops of the antigen-binding region [31]. To gain insight into the possible influence of the CDR H3 loop on C_60_ binding, we compared two models of the complex of C_60_ with the native form of FabC_60_ (Complex I) and with a modified model of FabC_60_ containing the CDR H3 with deleted Asp100 and Tyr101 residues (Complex II) (*Fig. 5*).

In Complex I, the C_60_ molecule binds to the surface of the antigen-binding site and forms π–π stacking interactions with residues Tyr50 (H2), Tyr101 (H3), and Trp93 (L3). C_60_ binding leads to a 40% decrease in the solvent-accessible area of the C_60_ hydrophobic surface. The AutoDock Vina-generated binding energy is -7 kcal mol-1, which corresponds to a dissociation constant (*K*d) of the complex equal to 7.4×10^-6^ M. The *K*d constant experimentally determined earlier for the C_60_ complex with full-length anti-fullerene antibody was 1.1 × 10^-7^ M [18]. Taking into account the 80-fold decrease in the affinity of interaction between C_60_ and FabC_60_, *K*d for the complex of C_60_ with FabC_60_ can be estimated at 9×10-6 M, which correlates well with the value of *K*d calculated for the docking-simulated model of C_60_ in a complex with the native form of FabC_60_.

The removal of the Asp100 and Tyr101 residues allows the C_60_ molecule in Complex II to penetrate deeper into the antigen-binding cavity and bind at a distance of about 4 Å from the position of C_60_ in Complex I (*Fig. 5*). The AutoDock Vina-generated binding energy increases from -7 to -12 kcal mol-1, which corresponds to a decrease in *K*d of the complex from 7.4 × 10-6 to 1.6 × 10-9 M. The increase in affinity in the absence of Asp100 and Tyr101 can be attributed to the formation of additional (apart from those mentioned above) π–π interactions with His35 (H1) and Tyr34 (L1) and also to a 40% decrease in the solvent-accessible area of the C_60_ hydrophobic surface. Thus, the Asp100 and Tyr101 residues of the CDR H3 can play a key role in the reduction of the affinity of interaction between C_60_ fullerene and the corresponding antibodies.

Earlier attempts to obtain a complex between the antibody and fullerene were unsuccessfull [15]. The complex was simulated with INSIGHT 2 by a procedure different from that used in our work: the C_60_ molecule was manually placed into the cavity of the variable domain between the H and L chains, followed by minimization of the energy of the system [15]. Binding of C_60_ to anti-C_60_ Fab was ensured by interactions with the Tyr36, Gln89, Phe96, and Phe98 residues of the L chain and the Asn35, Trp47, and Trp106 residues of the H chain. Binding was accompanied by 90% reduction in the solvent-accessible area of the fullerene hydrophobic surface. At the present time, the structures of the complex of C_60_ with a synthetic protein (pdbid 5hkn, 5hkr, 5et3) [32] are the only experimentally established structures that can be used to verify the validity of our conclusions about the driving forces for the formation of the antibody–fullerene complex. In these complexes, C_60_ fullerene was bound in the hydrophobic pocket. C_60_ binding led to a ~90% decrease in the solvent-accessible area of the C_60_ hydrophobic surface and formation of a π–π interaction with the Tyr9 residue. The analysis of the structures performed in the present study revealed a π-π interaction between C_60_ and the Leu19-Ala20 peptide bond that was not mentioned in [32].

## CONCLUSION

In summary, the docking simulation data obtained in this study are in agreement with the experimental results [32]. Thus, π–π stacking interactions between fullerene and aromatic residues of the antigen-binding site and reduction in the solvent-accessible area of C_60_ make the defining contribution to the formation energy of the fullerene–antibody complex. A fragment of the mobile CDR H3 loop located on the surface of FabC_60_ that hinders the access of C_60_ to the antigen-binding site is the key structural factor responsible for the low affinity of the antibodies under consideration for C_60_ (*K*_d_ is about 10^-7^ M).

The Thr33 and His35 residues of the antigen-binding pocket, which are probably involved in fullerene binding in the solution, formed hydrogen bonds with the C-terminal residues of the symmetry-related FabC_60_ molecule under the crystallization conditions used, thereby stabilizing the crystal packing of the free form of FabC_60_ and interfering with the crystallization of the complex formed by C_60_ and FabC_60_.

## Abbreviations

STI: soybean trypsin inhibitor
TG: thyroglobulin
TMB: 3,3´,5,5´-tetramethylbenzidine
PBS: phosphate buffered saline
PBST: phosphate buffered saline supplemented with 0.05% Triton X-100
CDR: complementarity-determining region
FabC60: Fab-fragment of anti-fullerene C60 antibody
Kd: dissociation constant
r.m.s.d.: root mean square deviation
SolC60: fullerene aminocaproic acid
